# Adult-Onset Still’s Disease: Novel Biomarkers of Specific Subsets, Disease Activity, and Relapsing Forms

**DOI:** 10.3390/ijms222413320

**Published:** 2021-12-11

**Authors:** Beatrice Maranini, Giovanni Ciancio, Marcello Govoni

**Affiliations:** Rheumatology Unit, Department of Medical Sciences, University of Ferrara, 44121 Ferrara, Italy; g.ciancio@ospfe.it (G.C.); gvl@unife.it (M.G.)

**Keywords:** Still’s disease, AOSD, biomarkers, refractory, relapsing

## Abstract

Adult-onset Still’s disease (AOSD) is a systemic inflammatory disease of unknown etiology. Recent studies have demonstrated that the hallmark of AOSD is a cytokine storm, which is characterized by the excessive production of interleukin (IL)-1, IL-6, IL-18, tumor necrosis factor-α (TNF-α), and interferon-γ (IFN-γ), suggesting how pro-inflammatory cytokines play an important role in the pathogenesis of this disease. Actually, a certain proportion of patients (around 17–32%) with severe clinical symptoms achieves only partial remission or is resistant to both first-line corticosteroids and second-line DMARDs. These patients are defined as refractory AOSD patients, requiring higher dosage glucocorticoids, longer treatment duration, or the simultaneous introduction of immunosuppressive drugs, further leading to AOSD relapses. In this narrative review, we will analyze the latest literature data to unravel potential pathogenetic factors associated with specific patterns of AOSD disease or relapses in order to identify biomarkers that may guide clinical decisions, eventually leading to new therapeutic options.

## 1. Introduction

Adult-onset Still’s disease (AOSD) is a rare systemic inflammatory disease of unknown etiology characterized by high intermittent fever, an evanescent salmon pink skin rash, arthritis, and increased acute phase reactants, frequently accompanied by myalgias, sore throat, lymphadenopathies, splenomegaly, and neutrophilic leukocytosis [1,2,3]. It is more common in females with the mean age usually ranging from 16 to 35 years, although onset at older ages has been observed as well [1,3].

AOSD has recently been considered to be an autoinflammatory disease due to phenotypical signatures and the absence of a significant increase in autoantibody levels [4], although AOSD has negative genetic testing in family histories, which is opposite to other autoinflammatory conditions [5].

The exact pathogenic mechanisms of the disease are only partially understood. Our understanding of the mechanisms underlying AOSD pathogenesis is mostly hypothetical, but studies suggest the involvement of a pro-inflammatory cascade. The starting point of the pro-inflammatory cascade is probably danger signals such as pathogen-associated molecular patterns (PAMPs) or damage-associated molecular patterns (DAMPs). Then, these danger signals are transmitted to macrophages and neutrophils through specific Toll-like receptors (TLRs) that activate inflammasomes, leading to caspase activation and the overproduction of pro-inflammatory cytokines, such as IL-1β, IL-6, IL-8, IL-17, IL-18, and TNF [6,7].

The disease may be life-threatening, leading to death because of severe complications, including macrophage activation syndrome (MAS), cardiopulmonary system involvement, and fulminant hepatitis [8]. Recent studies have demonstrated that the hallmark of AOSD is a “cytokine storm”, which is characterized by the excessive production of interleukin (IL)-1, IL-6, IL-18, tumor necrosis factor-α (TNF-α), and interferon-γ (IFN-γ), suggesting that pro-inflammatory cytokines play an important role in the pathogenesis of this disease [9,10]. Current treatments include non-steroidal anti-inflammatory drug (NSAIDs) and glucocorticoids as the first-line therapy, which is followed by conventional and biological disease-modifying anti-rheumatic drugs (DMARDs) for more severe cases [11,12,13,14]. A certain proportion of patients (around 17–32%) with particularly severe clinical symptoms achieves only partial remission or is resistant to both first-line corticosteroids and second-line DMARDs [15]. These patients are defined as refractory AOSD patients, and they often require higher dosage glucocorticoids, longer treatment duration, and the simultaneous introduction of immunosuppressive drugs, including bDMARDs [3,8].

AOSD may present and evolve with different courses. Cush et al. [16] classified AOSD in 1987 as having three clinical main patterns: namely, polycyclic disease, chronic articular disease, and monocyclic disease with different prognosis and outcomes. However, the identification of factors predicting poor outcomes is still lacking [17]. In addition, only few studies have addressed the prognostic factors of AOSD [18].

In this narrative review, we analyzed more recent literature data to unravel potential pathogenetic factors associated with specific patterns of AOSD disease course or relapses, in order to identify biomarkers that may guide clinical decisions and new therapeutic options, too.

To ensure the present review provided a comprehensive update on recent developments in this field, search strategies were adopted in accordance with recommendations for narrative reviews [19]. Data were identified within the National Institutes of Health’s National Library of Medicine (PubMed) and Embase up to 7 August 2021. AOSD, Still’s disease, biomarkers, refractory, relapsing, disease activity, and their respective MESH terms were used as keywords. Only studies published in the English language were included, and the additional references quoted in these articles were also checked. Both basic and clinical studies were selected.

## 2. Gene Polymorphisms

The involvement of autophagy in AOSD pathogenesis has been suggested based on the finding that both elevated levels of autophagosome formation and autophagy-related gene (ATG) expression positively correlated with disease activity [20]. In their recent study, Hung et al. [21] investigated the associations of ATG polymorphisms with AOSD susceptibility, clinical manifestations, and disease course by comparing the variations of ATGs between patients with AOSD and healthy controls.

Significant linkage disequilibrium was noted in three single nucleotide polymorphisms (SNPs) of the autophagy-related 16-like 1 (*ATG16L1*) gene, which is involved in extending phagophores through the ATG5–ATG12–ATG16L1 complex during autophagosome formation [22]. In particular, the AA/CC/TT haplotype of ATG16L1 was associated with lower levels of autophagosome formation, a lower proportion of arthritis, and a significantly higher proportion of skin rash and systemic patterns of AOSD compared with other haplotypes, while no association with the susceptibility of AOSD patients was observed. Interestingly, the polymorphisms in humanATG16L1 have been also associated with an increased risk of Crohn’s disease, which is another well-known autoinflammatory disorder [23,24].

Another important aspect in AOSD pathogenesis is the dysregulation of NLR3-containing a pyrin domain (NLRP3) inflammasome signaling. Growing evidence indicates that the NLRP3 inflammasome plays a pivotal pathogenic role in autoinflammatory diseases: pharmacological inhibition of NLRP3 activation results in potent therapeutic effects, as observed through good response to interleukin (IL)-1β inhibitors and IL-18 binding protein in AOSD patients [25].

A recent work of Hung et al. [26] aimed to investigate the associations of genetic polymorphisms of NLRP3-inflammasome signaling with AOSD susceptibility and disease outcome. Among 66 AOSD patients, 53 SNPs were candidate and genotyped as involved in the NLRP3-inflammasome response. Functional assays showed that serum CARD8 levels were significantly lower in AOSD patients compared to healthy controls, while levels of caspase-1, IL-1β, and IL-18 were significantly higher. Moreover, the authors identified a novel genetic variant, the SNP rs11672725 of the CARD8 gene, as a significant genetic variant for AOSD susceptibility, accordingly to what was already known: that CARD8 is a negative regulator of NLRP3-inflammasome signaling, since it is negative regulator of NF-kB. A major limitation of this study relies on the enrollment of Chinese patients only and not other ethnic groups, restricting generalizations of these findings, requiring further studies to identify genetic variants. 

## 3. The Role of Ferritin

Ferritin has a strategic role in the pathogenesis of AOSD. It is an intracellular iron storage protein including 24 subunits that are categorized, according to molecular weight, as heavy (FeH) subunits and light (FeL) subunits.

In a recent retrospective study performed in Japan [27], serum ferritin and heme-oxygenase 1 (HO-1) levels were assessed in 110 AOSD patients and 46 controls. Results revealed that serum ferritin and HO-1 levels were significantly higher in active and relapsed AOSD cases and decreased during treatment. Thus, if confirmed in further studies, serum HO-1 could serve as strong predictors of disease relapse and therapeutic response, too [3]. 

A recent work by Kim et al. [28] aimed to elucidate the value of combination of various laboratory inflammatory scores, including the systemic immune-inflammation index (SII), C-reactive protein/albumin ratio (CAR), albumin/globulin ratio (AGR), prognostic nutritional index (PNI), and ferritin/erythrocyte sedimentation rate ratio (FER) as assessment factors for the diagnosis and evaluation of disease activity in AOSD. The study is relevant, since it proposes a novel simple score, derived from a combined index (SII + ferritin). In fact, SII + ferritin positively correlated with all disease activity indices except hemoglobin; all correlations other than those of the CAR and CRP, the FER and ferritin, and SII + ferritin were placed between weak to moderate. As SII + ferritin has not been tested previously, future studies are needed to validate its diagnostic utility. 

## 4. Cellular Signaling Imbalance

Other studies investigated the cellular crosstalk in AOSD patients.

AOSD pathogenesis is characterized by circulating natural killer (NK) cells and their IFN-γ-producing ability. In the study of Shimojima et al. [29], NK cells and their IFN-γ expression levels were analyzed by flow cytometry in blood samples from AOSD patients. Additionally, cytokine receptors of IL-12, IL-15, and IL-18 on NK cells were also measured. As a result, NK cell count was significantly lower in acute AOSD than in healthy controls and significantly increased in remission AOSD patients. As expected, the expression of IL-12 and IL-15 receptors on NK cells was significantly increased in acute AOSD, while the IL-18 receptor was not significantly different among groups. IFN-γ expression in NK cells was significantly higher in acute AOSD than in healthy controls and significantly decreased in remission AOSD, suggesting that disease activity may be implicated in IFN-γ-expressing cells in AOSD. However, the absolute number of NK cells and IFN-γ-expressing NK cells revealed an inverse correlation with serum ferritin levels in acute AOSD, which is an observation that needs to be fully elucidated.

As already explained, neutrophilic leukocytosis is a key feature of AOSD. It is well-known that both the modulation of miRNAs and neutrophil extracellular traps (NETs) formation are implicated in inflammatory disorders. Accumulating evidence revealed NETs formation in AOSD patients, although the regulatory roles of miRNAs in NETs formation in AOSD remains unclear. Jia et al. [30] demonstrated how circulating the NETs signature provides added clinical value in monitoring AOSD patients, allowing predicting who is more prone to be refractory to low-dose glucocorticoids.

Collected observations in the literature led to hypothesizing that microRNAs (miRNAs) may play an important role in AOSD pathogenesis [31]. However, few data are available concerning the expression of circulating miRNAs in AOSD patients. Liao et al. [32] demonstrated an upregulation of circulating microRNA 134 (miR-134), which is associated with elevated IL-18 levels and with a positive correlation with disease activity. In a more recent study, Liao et al. [33] revealed how circulating levels of IL-18, NETs, and miR-223 were significantly higher in active AOSD patients compared with inactive AOSD patients or healthy controls. Moreover, IL-18 increased calcium influx into neutrophils, inducing mitochondrial ROS (mROS) production and NETs formation. Elevated levels of NETs-DNA increased miR-223 expression in neutrophils, and the upregulated miR-223 expression suppressed mROS production by blocking calcium influx, subsequently inhibiting IL-18-mediated NETs formation. In vitro assays demonstrated that neutrophil-derived small extracellular vesicles carrying miR-223 could repress IL-18 production in macrophages. Interestingly, the increased neutrophil-derived exosomal miR-223 levels were principally registered in active AOSD patients compared with healthy controls. Curiously, together, these results suggest a possible underlying mechanism between inflammatory (IL-18 induced NETs) and anti-inflammatory (miR-223) pathways in AOSD. Potentially, miR-223, mROS inhibitors, and calcium channel blockers may be addressed as therapeutic targets.

In recent years, among non-coding RNAs (ncRNAs), the more recently identified long ncRNAs (lncRNAs) have emerged as potential diagnostic and therapeutic biomarkers in both inflammatory diseases and infectious diseases [34,35,36,37]. Functional analysis on the targets of lncRNAs was also involved in the regulation of immune processes, inflammatory mediator regulation of Transient Receptor Potential (TRP) channels, lung cell differentiation, negative regulation of NF-kB-inducing kinase (NIK)/NF-kB signaling, and negative regulation of T cell differentiation, which all play significant roles in depressing viral infection [38].

In a recent study of Yang et al. [39], the authors developed an AOSD diagnostic scoring system based on the expression signature of lncRNAs MIAT, THRIL, and RMRP. Furthermore, they demonstrated that the combined expression signature of MIAT, THRIL, and RMRP could differentiate AOSD from systemic lupus erythematosus, rheumatoid arthritis, and potentially also sepsis. From the aspect of AOSD prognostication, the score showed interestingly a good specificity.

Other important molecules involved in AOSD pathogenesis are DAMPs, which mediate inflammasome activation. As widely known, along with extrinsic factors, intrinsic pathways can trigger an unexpected immune response [40]. DAMPs and PAMPs are recognized by Toll-like receptors, and they may induce the potential activation of innate immune cells, leading to inflammatory response in AOSD. Most DAMPs, which include nucleic acids, intracellular proteins, and extracellular matrix components, are released by damaged tissues or dying cells [41]. As well, PAMPs include microbial components such as lipopolysaccharides (LPS) from Gram-negative bacteria and viral single-stranded RNA. The expression levels of genes encoding DAMPs contribute to the susceptibility to AOSD. Therefore, the study of DAMPs and PAMPs reflect a particular interest in AOSD [40].

Cold-inducible RNA-binding protein (CIRP) belongs to a family of cold-shock proteins that react to cellular stress and has been identified as a DAMP trigger in inflammatory responses. A recent study of Fujita et al. [42] investigated the clinical significance of serum CIRP levels in AOSD and found significantly higher levels AOSD patients compared with RA patients and healthy controls. Additionally, there was a significant positive correlation between serum CIRP levels, AOSD disease activity score, as well as ferritin and IL-18 values, although no significant difference in serum CIRP levels was found among the three AOSD phenotypes (polycyclic systemic type, monocyclic systemic type, chronic arthritis type).

Another interesting aspect to investigate is the levels of checkpoint molecules, whose dysregulated expression has been reported leading to various inflammatory or autoimmune conditions. The interaction between galectin-9 (Gal-9) and its ligand, T cell immunoglobulin and mucin-containing-molecule-3 (TIM-3), one of the coinhibitory receptors, aims at transducing the inhibitory signaling to regulate immune responses. The results of the study of Fujita et al. [43] showed significantly higher serum Gal-9 levels and similarly sTIM-3 levels in patients with AOSD compared to patients with RA and healthy controls. In addition, serum levels of Gal-9 or sTIM-3 showed positive correlations with IL-18 levels, serum ferritin, and AOSD disease activity score, whereas there was no significant correlation between serum Gal-9 or sTIM-3 and CRP. Curiously, AOSD patients with the chronic arthritis phenotype had a significantly higher Gal-9/ferritin and sTIM-3/ferritin ratio than those with other phenotypes. Both Gal-9 and sTIM-3 levels showed a significant decline after immunosuppressive treatment, which is coherent with disease activity scores. 

Few studies have evaluated the role of Toll-like receptors as biomarkers of AOSD. Moreover, only a few studies have evaluated the utility of serum levels of their ligands as biomarkers, because cell surface TLRs levels are difficult to establish. A recent review by Jung et al. addressed these issues [40] and found how a number of TLR4 ligands (S100A8/A9, S100A12, MPO-DNA complex, high mobility group box 1 (HMGB1), and NET molecules), while contributing to inflammation, may be reliable biomarkers for evaluating disease activity in AOSD. In particular, the serum level of the TLR4 ligand S100A8/A9 seems to correlate with disease activity (based on acute-phase reactants and subjective patient assessments) [44].

## 5. Innate System Activation

Another important aspect in AOSD pathogenesis relies on the innate immune system activation, with an increased number of macrophages and neutrophils. In AOSD patients, serum levels of calprotectin, a calcium-binding protein released during the activation of neutrophils and macrophages, and soluble CD163, which is released by activated macrophages, are higher; up to now, only a few studies proved their levels to be correlated with disease activity [45,46].

Recently, another hallmark of chronic course of AOSD has been investigated: triggering receptor expressed on myeloid cells-1 (TREM-1) is an amplifier of inflammatory signals. A study of Wang et al. [47] found correlations between its soluble form (sTREM-1) levels and AOSD disease activity, clinical characteristics, and laboratory data. In this study, sTREM-1 levels were significantly elevated in AOSD patients compared to RA patients and healthy controls. Serum sTREM-1 levels correlated with the disease activity score, ferritin, leucocyte count, CRP, and pro-inflammatory cytokines, including IL-1 and IL-6, indicating that sTREM-1 contributes to discriminating patients developing a chronic course from patients developing non-chronic course. However, it must be kept in mind that to date, the natural ligands of TREM-1 have not yet been fully identified; possibly, HMGB1 represents a TREM-1 ligand, since a direct interaction between TREM-1 and HMGB1 was detected by immunoprecipitation and cross-linking assays [48]. To date, sTREM-1 has been reported to be a potential biomarker in several diseases; for instance, it has been proposed how sTREM-1 in bronchoalveolar lavage fluids may help clinicians diagnosing patients with bacterial rather than fungal pneumonia [49]. Although the exact functions of sTREM-1 remain to be defined, sTREM-1 is thought to negatively regulate TREM-1 signaling by neutralizing TREM-1-mediated inflammatory response [47]. 

## 6. Cytokine Storm

As evidenced by several recent studies, the excessive production of pro-inflammatory cytokines such as IL-1, IL-6, IL-18, tumor necrosis factor-α (TNF-α), and interferon-γ (IFN-γ) plays an important role in the pathogenesis of the disease [9,10].

Among these, the role of IFN-γ in AOSD remains controversial. It promotes pro-inflammatory responses, such as host defense responses and regulatory functions, including inhibition of neutrophil-specific chemokines and induction of T-cell apoptosis. However, signature IFN-γ-induced cytokines or chemokines, such as IL-18 or C-X-C motif chemokine 10 (CXCL10), are significantly higher in AOSD [50]. Han et al. [51] showed that CXCL10 levels are elevated in AOSD patients compared with rheumatoid arthritis (RA) patients and healthy controls, and these levels correlated with AOSD disease activity markers. A more recent work of Han et al. [52] showed that serum levels of CXCL9, CXCL10, and CXCL11 in patients with AOSD correlated with several inflammatory markers and systemic scores, while they decreased after improvement in disease activity consequent to treatment. Moreover, by immunohistochemistry, the percentage of CXCL10-positive inflammatory cells was higher in skin biopsy samples from AOSD patients than in those from healthy subjects, eczema, or psoriasis groups. These results support the hypothesis that IFN-γ-induced chemokines potentially play a relevant role in the inflammatory pathogenesis of AOSD and may be proposed as a biomarker of disease activity and response to treatment [52].

Additionally, the expression levels of CXCL10 and CXCR3 were revealed to be higher in histopathological lymph node samples of patients with AOSD than in patients with T cell lymphoma, histiocytic necrotizing lymphadenitis, tuberculous lymphadenitis, and reactive hyperplasia [53]. The aforementioned immunohistochemical staining is crucial to avoid improper diagnosis or treatment, aiding in differentiating AOSD from other mimickers.

In their study, Han et al. [54] showed that serum CXCL10 levels correlated with ferritin and systemic scores, while serum CXCL13 levels correlated with hemoglobin, CRP, ferritin, albumin, and systemic scores, further emphasizing the potential pathogenetic role of CXCL10 and CXCL13 in the pathogenesis of active AOSD. Moreover, in follow-up treated AOSD patients, the levels of CXCL10 and CXCL13 fell consistently [54].

Recently, attention has been given to IL-18 and the S100A8/A9 protein 1 [44,55,56,57]. Kudela et al. [57] aimed to clarify the role of IL-18 as a diagnostic and disease activity marker in AOSD. In their study, a significant elevation of IL-18 serum levels in active AOSD and systemic onset juvenile idiopathic arthritis (sJIA) was found in comparison with other rheumatic diseases, and a clear association of IL-18 serum levels with disease activity in AOSD was evidenced. These results support the potential role of IL-18 as an important biomarker in AOSD and sJIA. Furthermore, the normalization of IL-18 serum levels was observed in AOSD patients who are in remission, although the latter finding is in contrast with other previous publications, in which a decline of IL-18 in AOSD remission patients was not recorded [44]. Regarding the S100A8/A9 protein, in the study of Hyoun-Ah Kim et al. [44], serum levels of S100A8/A9 were higher in AOSD patients than in those observed in RA and controls and correlated with leukocyte count, erythrocyte sedimentation rate, C-reactive protein, ferritin, and systemic disease score. Moreover, S100A8/A9 levels decreased in AOSD patients achieving remission, suggesting that serum S100A8/A9 may be a useful biomarker for evaluating disease activity [44]. 

Among cytokine storm, IL-1β is thought to play a crucial role. IL-33, as a member of the IL-1 family and a ligand of the orphan receptor ST2, drives inflammatory pathways through innate and adaptive immunity. In their study, Han et al. [58] examined the associations between IL-33/ST2 levels and clinical manifestations of patients with AOSD and found that the levels of serum IL-33 and soluble ST2 were significantly elevated in patients with active AOSD compared with patients with RA or healthy controls; accordingly, serum IL-33 levels fell when disease activity declined during follow-up, after treatment. Moreover, serum IL-33 levels correlated with systemic score, ESR, ferritin levels, and aspartate transaminase levels. Differently, soluble ST2 levels in patients with AOSD were higher than those of healthy controls but comparable with patients with RA, and they correlated only with ferritin levels. The authors also confirmed that the expression of IL-33 and its receptor ST2 were elevated in rash material from patients with active AOSD compared to the skin of healthy controls. These results suggest that the IL-33/ST2 signaling pathway may play a consistent role in the pathogenesis of acute inflammation and particularly skin manifestations associated with AOSD [58]. 

Regarding articular involvement, a recent work of Ruscitti et al. [59] aimed at assessing the global transcriptomic profile of synovial tissues in AOSD. In this study AOSD patients underwent an MRI exam of joints to assess articular involvement patterns. Interestingly, patients with MRI-bone erosions showed a higher prevalence of splenomegaly, a more frequent chronic disease course, and lower levels of ESR and ferritin. Moreover, the AOSD synovial samples revealed a hyper-expression of IL-1, IL-6, and TNF, as well as their respective receptors, further reinforcing the rationale of targeting these cytokines through therapies.

## 7. Clinical Hallmarks

**Figure 1:**
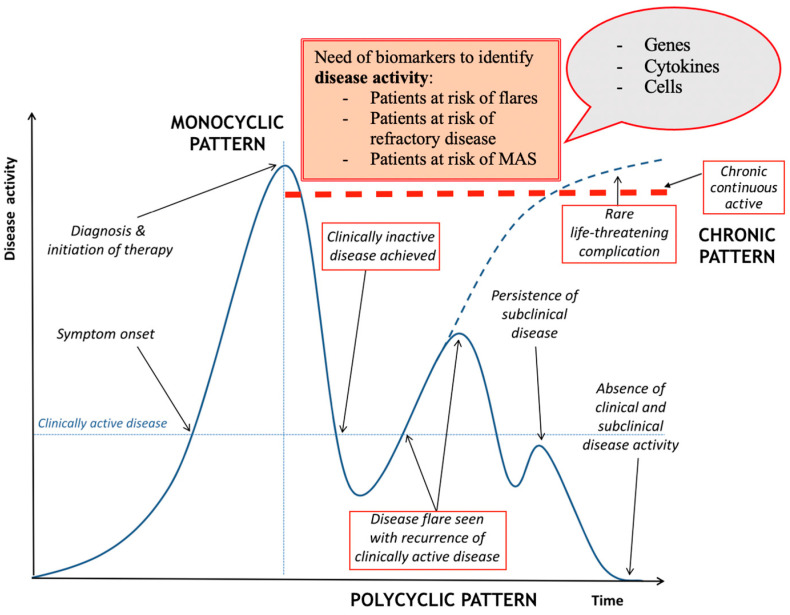
Representative clinical course of AOSD. Specific time points indicate when diagnostic and prognostic biomarkers are required. Diagnostic biomarkers have already been widely discussed, while prognostic markers are needed to detect disease activity, flares, and potentially unfavorable outcome. Modified from Mitrovic et al. [61].

Only a few studies in the literature have explored clinical factors associated with AOSD relapse, and to date, no clinical predictive models to early identify newly diagnosed patients at risk of having poor prognosis are available [60,61] (Figure 1).

Ruscitti et al. [62] employed system scores to correlate new disease outcomes to patients at a higher risk of mortality; however, mortality is not the first and most common event among the poor clinical effects associated with AOSD.

A recent study of Yin et al. [17] aimed to explore clinical indicators that may affect outcomes in a cohort of 174 AOSD patients to establish a well-differentiated prognostic nomogram in predicting the individual risk of long-term poor prognosis. Relapse/refractory disease was defined as a poor clinical outcome, which is characterized by the need of at least a 50% increase in glucocorticoid doses or a restart of glucocorticoid therapy. The clinical features, laboratory findings, and treatment regimen of all patients were recorded. Twenty-eight clinical factors were considered for the univariate logistic regression, including age, fever, disease duration, weight loss, platelet (PLT) count, serum ferritin, and erythrocyte sedimentation rate (ESR), which were proven to be statistically significant unfavorable factors for relapse/refractory AOSD. Prolonged disease duration from symptom to diagnosis represents a significant poor prognosis factor. An expected decrease in CRP after tocilizumab (TCZ) therapy was documented, since IL-6 is involved in CRP production. Therefore, the decrease in CRP may not be a reliable indicator of clinical remission. Surprisingly, in this study, elevated ESR is a favorable factor correlated with good prognosis, contrarily to other previous results [63]. As reported by Gerfaud-Valentin et al. and Giacomelli et al., fever led to a condition more related to the conversion into monocyclic AOSD [8,64], and a monocyclic disease course has been recognized as a poor prognosis factor. In several other reports, increased serum ferritin levels have been identified as an unfavorable factor, leading to the relapse of AOSD [65]. Interestingly, although the authors here failed to find any correlation between platelet count and AOSD prognosis, previous studies have shown that platelets also play a key role in inflammation [66].

Conversely, a study of Hung et al. [21] demonstrated that the occurrence of skin rash, arthritis, sore throat, lymphadenopathy, hepatosplenomegaly, leukocytosis, and disease activity scores, together with the levels of ferritin, IL-1, IL-6, IL-17A, IL-18, or TNF-α was not predictive of the systemic pattern of the disease nor of the clinical manifestations; furthermore, the above-mentioned biomarkers did not show any statistical significance prediction ability of a chronic articular pattern. These findings support the urgency to develop a rapid and efficient, but non-invasive, diagnosis method to improve targeted AOSD therapy.

The rapidly emerging field of metabolomics is a novel quantitative method of analyzing metabolite changes in a cell, tissue, or body fluids.

Metabolomics can be used to identify overall metabolic profiles, allowing the integration of the effects of genetic and environmental factors. Accumulating evidence suggests that the metabolite profile is altered in patients with rheumatic diseases [67]. Therefore, the identification of specific metabolomic profiles in rheumatic diseases has provided novel insights into disease mechanisms. Some authors hypothesized that metabolomics could be employed to detect metabolic biomarkers that may facilitate the diagnosis and disease outcome in AOSD [68]. 

Intriguing results have been provided by Sun et al. [69], who first performed a urinary proteomic study in AOSD patients. These authors found that the urinary proteins were enriched in pathways of the innate immune system and neutrophil degranulation, detecting α-1-acid glycoprotein 1 (LRG1), orosomucoid 1 (ORM1), and ORM2 proteins as highly expressed in AOSD patients compared to healthy subjects. Furthermore, these parameters were positively correlated with disease activity score, transaminases values, ESR, and cytokines levels, most of all IL-1β, IL-6, and IL-18. This study is the first to identify possible novel urinary markers for the non-invasive and simple screening of AOSD activity.

In a retrospective observational study carried out at our tertiary-referral Rheumatology Unit on seventy-six AOSD patients, ferritin level as well disease activity score (DAS(28)) were associated with the rate of progression of the articular manifestations of the disease, and a polyarthritis persisting over 6 months was associated with the development of a chronic articular course, irrespective of the size of the involved joints. On this basis, we concluded that ferritin with DAS-28 might serve as a useful predictor for a progressive chronic course of the disease, as measured with a simple erosion narrowing score [18].

In Table 1, we reported the main relapsing/refractory AOSD biomarkers reported in the literature so far.

## 8. Biomarkers for Differential Diagnosis

Dealing with the global SARS-CoV-2 pandemic, a study of Chen et al. [70] given the similar clinical features of COVID-19 and AOSD tried to identify possible biomarkers capable of differentiating between these two diseases. A phenomenon observed in COVID-19 patients is NLRP3-inflammasome activation leading to the overproduction of IL-18 [71], which is similar to that observed in AOSD. In the study of Chen, as already proved, among the cytokines involved, IL-18 acted as the most significant predictor of active AOSD. Moreover, active AOSD patients had 68-fold higher levels of IL-18 than severe SARS-CoV-2 infected patients. In addition, IL-18 showed the highest discriminating ability between AOSD and COVID-19 in the ROC analysis of the putative markers. Thus, blocking IL-18 has therapeutic efficacy for AOSD but has yet to be applied to COVID-19 treatment now.

Another important aspect is that, given the similarities in clinical and laboratory features between AOSD and sepsis, and the divergent treatment strategies for these two conditions, specific biomarkers are crucial for a nearly correct differential diagnosis. A recent study of Tian et al. [72] found that serum heparin-binding protein (HBP) levels were significantly higher in patients with active AOSD rather than in those with inactive AOSD; however, patients with sepsis showed higher serum HBP levels compared with the AOSD group, even in the active disease group. This study supports HBP as a useful diagnostic biomarker to evaluate disease activity in patients with AOSD and to differentiate AOSD from sepsis. Similar results were obtained before by Zhou et al. [73], who found that the area under the ROC curve (AUC) of HBP was significantly higher than those of procalcitonin and CRP, demonstrating once more again the relevance of this hallmark. 

## 9. Biomarkers of Organs’ Damage

Another important aspect lies in the predictive role of certain biomarkers to identify patients at high risk of poor prognosis, in order to better predict complications. A study of Di Benedetto et al. [74] in a cohort of 147 patients found how serum levels of ferritin and CRP, at the time of diagnosis, correlated with an increased risk of MAS and mortality, respectively, suggesting the possibility of an early detection of aggressive subsets at higher risk of complications. Considering that CRP and ferritin values are widely available in clinical practice, these results may be readily transferable into daily practice, thus balancing an appropriate escalation of therapy and improving the management of patients with AOSD.

Liver damage is another important complication of AOSD. The activation of neutrophils may hesitate in the infiltration of liver and is suspected to promote tissue injury. A study of Jia et al. [75] aimed at identifying candidate biomarkers associated with liver damage in AOSD. A transcriptome analysis of neutrophils from treatment-naive active AOSD patients and healthy controls was performed. Neutrophils from active AOSD patients showed a very strong neutrophil degranulation process, with Lipocalin-2 (LCN2) as one of the top five upregulated granule genes. LCN2 expression was assessed in neutrophils as well as in plasma and liver biopsies of AOSD patients. The authors identified LCN2 as one of the five most significantly upregulated genes encoding granule proteins in AOSD neutrophils compared with healthy controls, RA, systemic lupus erythematosus, or liver injury by other causes. Plasma LCN2 levels were strictly correlated with inflammatory markers, disease activity score, and cytokines levels. LCN2 levels were also found to be increased in active AOSD with liver involvement and independently associated with liver dysfunction. The authors further demonstrated elevated levels of LCN2 in liver biopsies from three patients with ongoing liver injury. Therefore, LCN2 provides evidence as a potent biomarker for identifying systemic involvement AOSD and especially liver damage caused by hyperinflammation. Further elucidations are needed to identify the role of LCN2 in early stages of hepatic inflammation.

Further interesting results derived from the work of Liu et al. [76], who explored whether receptor-interacting serine/threonine kinases (RIPKs) in lymphocytes could affect the pathogenesis of AOSD, especially considering liver involvement. To this aim, they studied liver damaged AOSD and non-liver damaged AOSD patients and found that in AOSD patients, there was an abnormal lymphocytes distribution; furthermore, disease activity positively correlated with the percentage of CD3+ T cells. Patients with liver involvement were younger and showed a higher disease activity score than patients without liver involvement, with higher frequencies of CD3+ T cells, especially higher CD8+ T cells. An aberrant expression of receptor interacting serine/threonine kinases (RIPKs) in lymphocytes was documented in AOSD patients compared to healthy controls, showing significantly higher RIPKs expression in patients with liver involvement than in patients without liver damage. In addition, RIPKs positively correlated with ESR and disease activity in AOSD. As RIPKs were correlated with ESR and disease activity score, they can be applied to monitor AOSD disease activity.

## 10. Ongoing Trials

Owing to the rarity of the disease, well-designed prospective studies in AOSD patients are limited; furthermore, there are very limited randomized controlled trials (RCTs) in AOSD patients evaluating management strategies.

Only few country-specific guidelines for the management of AOSD have been published, and a treat-to-target approach is still lacking. As already mentioned, the current AOSD treatment paradigm includes NSAIDs and steroids as first-line therapy, conventional synthetic disease-modifying anti-rheumatic drugs in steroid-refractory patients, and biologics in those resistant to conventional treatment. 

Anakinra, a recombinant IL-1 receptor antagonist, was the first biologic molecule to show beneficial effects in treating systemic and articular manifestations of AOSD in many case series, uncontrolled trials, and different national surveys [77]. Although the evidence for the efficacy of anakinra is unproven in classical double-blinded controlled trials, the overall number of published cases (>250) provided convincing evidence [7].

In an RCT among AOSD patients, the treatment of active AOSD with canakinumab led to improvements in several outcome measures, which were defined as a reduction in disease activity at week 12 of treatment [78]. Canakinumab is the only FDA-approved bDMARD for the treatment of AOSD, while in Europe, both canakinumab and anakinra are approved [3].

Other biologics including tocilizumab, abatacept, rituximab, and tofacitinib have also been investigated in AOSD. A randomized, double-blind, placebo-controlled phase III trial suggested that tocilizumab is an effective therapy in AOSD, although the primary endpoint was not met [79]. Therefore, tocilizumab should be considered as an alternative to IL-1 antagonists, particularly when an articular subset is present [64]. Abatacept (a modulator of T-lymphocyte activation) and rituximab (a monoclonal anti-CD20 antibody) have been assessed in refractory AOSD patients with little proven benefit [80]. A recent case report concluded that application of the Janus kinase (JAK) inhibitor tofacitinib in refractory AOSD patients contributed to disease remission and steroid sparing, especially in patients with polyarthritis [81].

As already explained, higher levels of free IL-18 are documented in some studies in patients with active disease compared with inactive disease, indicating that IL-18 may be a potential biomarker for evaluation of disease activity in AOSD. Tadekinig alfa is a recombinant human IL-18 binding protein. Recently, a phase II, open-label clinical trial examined for the first time the safety and efficacy of IL-18 blockade in patients with AOSD [82]: 23 refractory AOSD patients were recruited, presenting with fever or CRP levels greater than 10 mg/l. Patients received 80 mg or 160 mg tadekinig alfa three times per week. The levels of free IL-18 in serum dropped to almost undetectable levels within 2 h after injection and continued to be low up to 48 h.

It is very important to measure disease activity accurately during long-term follow up, but currently available biomarkers have limited value to predict the variable course of AOSD. 

As above-mentioned, acute phase reactants including C-reactive protein and erythrocyte sedimentation rate, serum ferritin levels, calprotectin, and free IL-18 were documented as useful biomarkers in clinical practice, even if with relatively small numbers of patients’ cohort. However, these parameters may not accurately depict disease activity, especially in patients who are treated with glucocorticoids and/or DMARDs; moreover, their real values and cost-effectiveness in clinical practice have not been validated yet. 

The development of composite biomarkers to detect disease activity could support rational decision making in the treatment of AOSD [83]. 

## 11. Conclusions

In the past few decades, substantial advancement has been made on AOSD etiology and its diagnostic biomarkers. However, to date, no reliable prediction hallmarks of disease course and treatment response have been established yet. The main problem often seen while studying biomarkers in AOSD is the small size of cohorts, which confers low statistical power when results are analyzed. Still, many recent insights in AOSD pathogenesis have been obtained and presented in this narrative review, from genetic association, involvement of the innate immune system, and the cytokine cascade. There is a pressing need to confirm and consolidate findings from discovery studies in order to validate biomarkers for clinical assessment.

Conceivably, the upcoming contributes will probably allow a better understanding of the disease course and its complications, driving to a better clinical management among its life-threatening manifestations, identifying new promising therapeutic targets, hopefully improving the outcome of AOSD in the near future.

## Figures and Tables

**Table 1:** Main prognostic clinical hallmarks (clinical features and laboratory findings) identified in the literature as predictive of relapsing/refractory AOSD.

Relapsing/Refractory Biomarkers	Conflicting Results
Age [17]	Erythrocyte sedimentation rate (ESR) [17,63], platelet (PLT) count [17,65,66]
Prolonged disease duration [17]	Skin rash, arthritis, sore throat, lymphadenopathy, hepatosplenomegaly [21]
Weight loss, fever, monocyclic disease course [8,17,64]	Leukocytosis [21]
DAS28 values [18]	Disease activity score based on Pouchot’s score [21]
α-1-acid glycoprotein 1 (LRG1), orosomucoid 1 (ORM1) and ORM2 levels [69]	Levels of ferritin [27,28,65], IL-1, IL-6, IL-17A, IL-18 and TNF-α [21]

## Data Availability

Data is contained within the article.
